# Single-Cell Omics in Legumes: Research Trends and Applications

**DOI:** 10.3390/plants14233615

**Published:** 2025-11-27

**Authors:** Yaohua Li, Md Sabbir Hossain, Marc Libault

**Affiliations:** Division of Plant Science and Technology, Interdisciplinary Plant Group and Christopher S. Bond Life Sciences Center, University of Missouri-Columbia, Columbia, MO 65211, USA; ylvy9@missouri.edu (Y.L.);

**Keywords:** single-cell transcriptomics, cell identity and regulatory networks, comparative cell atlas, nitrogen fixation, synthetic biology, translational breeding, legume crops

## Abstract

Legumes are important food crops and play a central role in sustainable agriculture through their ability to form symbiosis with rhizobia, soil bacteria that fix atmospheric nitrogen. Recent advances in single-cell and spatial transcriptomics, along with single-cell epigenomics, have enabled high-resolution analysis of gene expression dynamics and the prediction of cell-type-specific regulatory networks. In this review, we highlight recent progress in the use of single-cell omics in legumes, with a particular focus on how genes functioning in distinct cell types contribute to plant development, responses to pathogens, stress-induced plasticity, and the establishment of root nodule symbioses. Case studies in *Medicago truncatula*, *Lotus japonicus*, *Glycine max*, and *Arachis hypogaea* illustrate the shift from bulk to single-cell multi-omics. We conclude by outlining current limitations and future directions for building integrated legume cell atlases that will support translational research and crop improvement.

## 1. Introduction

Understanding the diversity and specialized functions of the cells composing legume plants and their specific transcriptome are essential for linking gene activity to plant development, stress adaptation, and symbiotic interactions. For instance, legumes uniquely engage in root nodule symbiosis with rhizobia, forming specialized root organs that harbor nitrogen-fixing bacteroids and, at the same time, offer a tractable model to study plant cell differentiation and organogenesis [1]. Beyond their ecological significance, legumes also provide powerful models to investigate organ development, plant–microbe signaling, and adaptive plasticity under stress. Advances in single-cell and spatial transcriptomics now allow researchers to dissect cellular heterogeneity across diverse tissues, including roots, leaves, seeds, pods, and nodules at single-cell resolution [2,3,4,5]. These approaches enable cell-type-specific functional annotation and comparative genomics across species, thereby broadening the scope of legume research beyond what bulk transcriptomics can achieve.

Recent advances in single-cell and spatial transcriptomics have enabled legume research by capturing rare cell types, transient states, and mapping gene regulatory programs at high precision [2,3,4,5]. These approaches reveal cell-type diversity, developmental trajectories, and spatially organized transcriptional programs that bulk RNA-seq could not resolve [6,7]. Applications include single-cell omics on *Lotus japonicus* (hereafter referred to as Lotus) root tips [8], various soybean organs [4,5,9], and studies of stress and pathogen responses [10] as well as root nodule symbiosis [2,11,12], including single-nucleus spatial maps [13], and functional insights [14]. Together, these studies provide a basis for redefining legume cell-type classification and enabling cross-species comparisons, while laying the groundwork for predictive modeling and translational breeding strategies.

This review synthesizes recent advances in legume single-cell biology, drawing on literature collected from PubMed and Web of Science up to August 2025 using the keywords “single-cell RNA-seq,” “spatial transcriptomics,” and the names of legume species. Peer-reviewed studies were prioritized, and recent preprints were considered when they provided new insights. It integrates multi-omics data from model and crop legumes, including *Medicago truncatula* (hereafter referred to as Medicago), Lotus, *Glycine max* (soybean), and *Arachis hypogaea* (peanut), as well as spatial transcriptomics studies. These representative legumes were selected as models to study biological nitrogen fixation, and because of their substantial agronomical value as major suppliers of plant-derived oil and protein. Specifically, soybean meal’s dry mass contains 40 to 49% of proteins, significantly contributing to the share of proteins used in global livestock feed [15]. Besides, soybean is the second most productive vegetable oil crop after oil palm, generating approximately 68 million tons of oil annually and accounting for nearly 28% of the global vegetable oil market, despite its seeds containing only about 20% oil [16]. This review highlights how these approaches have redefined cell-type classification, developmental trajectories, and gene regulatory programs. It also outlines methodological considerations and future priorities for cross-legume species integration, epigenomic context, and functional validation aimed at legume improvement.

## 2. From Bulk to Single-Cell Resolution: Building Cell-Level Insights

### 2.1. Findings Revealed by Bulk RNA-seq

Transcriptomic technologies have transformed plant biology over the past two decades. Early microarray studies [17] provided the first large-scale gene expression profiles including for the model legume Medicago [18] followed by RNA sequencing (RNA-seq) [19], which rapidly became the standard due to its greater sensitivity and resolution [20,21,22]. This technology provides a genome-wide view of gene expression from a biological sample, often from entire organs. By identifying genes that are up- or down-regulated in response to developmental cues or environmental stimuli, RNA-seq analyses uncover the role of plant genes that drive biological processes. As a result, RNA-seq has been widely used in legumes to study genetic programs controlling development, nutrient composition, biotic and abiotic stress responses, and symbiotic nitrogen fixation [23]. The first RNA-seq study in *Glycine max*, published in 2010, profiled root hair-specific transcriptomes following *Bradyrhizobium japonicum* infection and revealed rapid, genome-wide transcriptional reprogramming in root hair cells upon bacterial contact [24]. Since then, advances in sequencing technologies and decreases in cost have enabled the generation of numerous transcriptomic studies across diverse legume organs, which collectively uncovered the transcriptional reprogramming of a wide range of biological processes, including nodulation, organ development, and response to biotic and abiotic stress (e.g., drought, flooding, salinity, heat) [20,21,25,26,27,28,29,30,31,32,33]. For instance, Xu et al. [33] identified strong induction of ABA-responsive transcription factors (TFs), ROS-scavenging systems, and osmoprotectant biosynthesis pathways during drought, whereas Liu et al. [29] revealed substantial transcriptional reprogramming under salt stress affecting photosynthesis, ion transport, and phytohormone signaling pathways. Bulk RNA-seq has also yielded important insights into seed biology. For instance, transcriptomes generated across soybean seed developmental stages revealed the coordinated induction of sucrose transporters, lipid biosynthesis enzymes, and storage protein genes, helping define the molecular transitions underlying seed filling and composition [21,30]. Similarly, numerous bulk RNA-seq studies have been conducted in Medicago, Lotus, peanut, *Pisum sativum* L., and *Cicer arietinum* L. to identify key genes, transcriptional regulators, and adaptive mechanisms associated with tissue development, symbiotic interactions, and responses to biotic and abiotic stresses [34,35,36,37,38,39,40,41,42,43,44,45,46]. These datasets have greatly expanded the gene expression resources available for legumes, leading to the development of specialized online platforms such as SoyBase [47], LegumeInfo [48], Lotus Base [49], MtExpress [50], and PeanutBase [51] that provide centralized access to legume transcriptomic data.

### 2.2. Limitations of Bulk RNA-seq in Resolving Cellular Heterogeneity

Despite its value, bulk RNA-seq only captures average gene expression across heterogeneous cell populations, lacking the resolution to dissect transcriptional activity at the single-cell level. Given that plant tissues comprise diverse cell types, each with distinct transcriptional programs, there has been a growing need for technologies capable of resolving gene expression in individual cells and cell to cell communication. To bridge this gap, early approaches isolated specific plant cell populations using techniques such as fluorescence-activated cell sorting (FACS) and laser capture microdissection (LCM), followed by microarray or bulk RNA-seq analysis. LCM-based transcriptomics has also been successfully applied in legumes to study gene expression in specific tissues [52]. For example, in Medicago, Limpens et al. [53] and Roux et al. [54] utilized LCM to resolve gene expression in spatial zones of nodules, shedding light on nodule development and function. Bulk RNA-seq fails to capture the expression dynamics of individual cells, particularly rare cell types, spatial and temporal variation, and low-abundance transcripts such as TFs. To support the benefit of snRNA-seq, we recently reported that diverse nodule cell types (cortical, infected, and vascular) exhibit distinct transcriptional programs that bulk RNA-seq cannot resolve [2]. Similarly, bulk approaches fail to capture dynamic transitions among sub-cell types, such as the functional differentiation of three endosperm subpopulations described by Zhang et al. [4]. Another major limitation of bulk RNA-seq is its inability to reveal the transcriptome of rare cell types. For example, in *Arabidopsis thaliana* (hereafter referred to as Arabidopsis) the transcriptome of the quiescent center (QC) cells, a cell type that constitutes only a very small fraction of root cells, was established using a single-cell RNA-seq approach [55]. Such a result could not be achieved using a bulk RNA-seq strategy, where the expression profiles of QC cells are largely masked by the transcriptome of other cell types. In summary, although bulk RNA-seq has generated foundational knowledge in legume biology and continues to be a valuable tool, it falls short in revealing cell-type-specific transcriptional programs and those associated with rare or transient cell states, spatial heterogeneity, and dynamic developmental transitions. These limitations underscore the need for single-cell RNA sequencing technologies, which enable transcriptomic profiling at the single-cell level and allow researchers to uncover the cellular diversity and regulatory complexity hidden within bulk measurements.

### 2.3. Timeline of the Use of Single-Cell/Single-Nucleus RNA-seq in Legume Research

**Figure 1:**
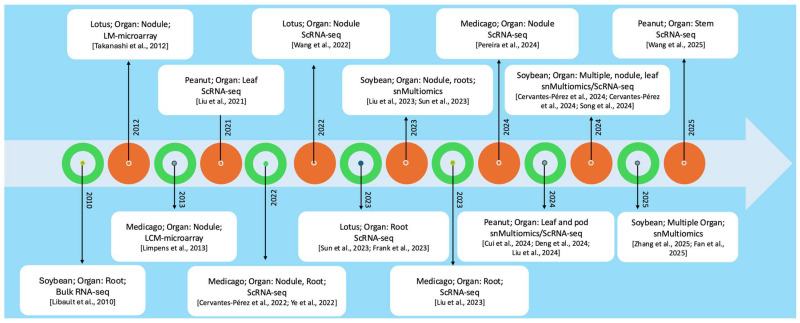
Timeline of key milestones towards single-cell transcriptomics research in legumes. (Note: References in this figure follow the numbering sequence of their first appearance in the main text to maintain consistency with the journal’s citation style) [2,4,5,8,9,10,11,12,13,14,24,52,53,57,58,59,60,61,62,63,64,65].

Today, the advent of droplet-based microfluidic single-cell/single-nucleus RNA sequencing (sc/snRNA-seq) technologies has revolutionized plant transcriptomics by enabling the profiling of thousands of individual cells in a single experiment at reduced cost. Unless otherwise specified, “scRNA-seq” hereafter broadly refers to both sc/snRNA-seq approaches. This approach utilizes cell barcoding and unique molecular identifiers to accurately assign transcripts to their respective cells of origin, while minimizing amplification biases. First demonstrated in Arabidopsis roots [55,56], droplet-based workflows have since been applied to various plant species and tissues, facilitating cell clustering, trajectory inference, and gene regulatory network reconstruction. In legumes, the first single-cell transcriptome was conducted in peanut leaf, identifying eight cell clusters with distinct transcriptional regulation related to leaf development [57]. Since then, scRNA-seq has been increasingly applied to legume species, including soybean, Medicago, peanut, and Lotus, covering both the early stages of rhizobial infection as well as analyses across different organs [2,4,5,8,9,10,11,12,13,14,24,52,53,57,58,59,60,61,62,63,64,65] (Figure 1). These advances have rapidly expanded legume transcriptomics research, providing cell-resolved insights into gene expression and cell biology. Beyond profiling the transcriptome of diverse and rare cell types, sc/snRNA-seq reveals pseudo-temporal changes in gene activity during development and stress responses. For example, Medicago nodules comprise meristematic, infection, nitrogen-fixation, and senescence zones [66], each characterized by distinct gene expression programs, unique chromatin organization, and histone mark status essential for their specific functions [54]. The emergence of scRNA-seq technologies has facilitated access to single-cell and cell-type transcriptomic resolution, enabling the precise capture of expression patterns of individual cells and cell types, including populations of infected and uninfected cells within Medicago nodules [59]. To further estimate the dynamic and continuous transcriptomic changes occurring in response to environmental and developmental cues, scRNA-seq enables the computational construction of cellular “trajectories” as cells transition between developmental states (e.g., during soybean embryogenesis, parenchyma cell differentiation in the peanut shell, and epidermal cell differentiation in peanut) [4,62]. Such analysis reveals transient populations, a more precise timing of gene regulation that drives dynamic processes and supports a more accurate inference of gene regulatory networks (GRNs). The latter is an important feature of scRNA-seq analysis, considering that bulk datasets may generate false positives (e.g., genes appear co-expressed but are active in different cell types) and false negatives (e.g., regulatory relationships are masked due to low expression in a small subset of cells).

### 2.4. Implementation of Single-Cell Multi-omics Strategies to Predict Gene Regulatory Networks

Single-cell transcriptomic technologies have significantly advanced our understanding of cellular diversity, yet they offer only a partial view of the complex regulatory mechanisms that define cellular states, which represent the dynamic variations within a single cell type driven by developmental cues or environmental conditions [67]. An integrative approach that captures multiple layers of information is needed to gain a more comprehensive understanding of plant cell biology, including their cell states. Combining scRNA-seq with single-nucleus Assay for Transposase-Accessible Chromatin using sequencing (snATAC-seq) enables simultaneous analysis of gene expression and chromatin accessibility at single-cell resolution, offering valuable insights into the epigenetic landscape that governs transcriptional regulation. Using this approach, approximately one-third of accessible chromatin regions (ACRs) have been identified as cell-type-specific, including many linked to phenotypic variations in Arabidopsis, maize, and soybean [4,68,69]. These findings underscore the potential of chromatin accessibility profiles as robust molecular markers of cell identity, comparable to or even more stable than gene expression signatures [70]. For example, in peanut, multi-omics analysis revealed the TFs interaction networks that regulate mesophyll and epidermal–guard cell differentiation. This study uncovered a core TF–fatty acid pathway module that modulates epidermal and guard cell identity, providing novel insights into epidermal cell differentiation. Notably, the expression of *WIP2* and *AP2* exhibited divergent patterns during epidermal expansion, while *KAN4* activity was found to respond to the oil synthesis pathway, mediating guard cell development [64]. In another peanut study, ATAC-seq identified 56 distinct vascular tissues-associated DNA motifs connected with pod development. Among them, aerial pegs were enriched for PIF/SGR motifs, whereas subterranean pegs showed HY5, HYH, and PIF1 motif enrichment. These light-responsive motifs highlight light-regulated transcriptional control as a key driver of pod vascular development. In the mature peanut pod, 36 distinct DNA motifs were identified, including 24 classified as ERF-related. This strong enrichment of ERF motifs suggests their key importance in driving the development and enlargement of the subterranean peanut pod [62]. In soybean, 13 sucrose transporter genes containing the DOF11 DNA-binding motif are co-upregulated during late peripheral endosperm development, a regulatory pattern that may likewise influence seed size and oil content. In soybeans, both molecular features (i.e., RNA- and ATAC-seq) have been independently captured at the single-cell level from various organs prior to their integration [4]. This approach necessarily triggers batch effects between independently processed samples. A solution to overcome this problem involves applying cell multi-omics technology, an approach that simultaneously captures both molecular layers from the same cell, linking chromatin states to transcriptional output and ultimately shaping cell behavior and fate decisions. Together, these integrative strategies support the identification of key regulatory elements and TFs that govern specific cellular states, ultimately advancing plant developmental biology and enabling precision strategies for crop improvement.

### 2.5. Integration of Spatial and Single-Cell Data

While single-cell transcriptomics has greatly advanced our understanding of cellular complexity, it cannot fully capture cell-to-cell interactions and tissue organization, and therefore, spatially organized biological processes involving different cell types that are fundamental to plant development, stress responses, and biotic interactions. Another limitation in non-model species, including legumes, is the limited availability of validated cell-type marker genes to support the accurate annotation of cell types based on their transcriptomic profiles. Homology-based methods for identifying markers often encounter difficulties due to gene duplication, neofunctionalization, or lineage-specific gene loss, which can lead to potential inaccuracies in defining cell types. To overcome these constraints, spatial transcriptomics has emerged as a complementary approach to scRNA-seq technologies. By preserving the spatial organization of tissues, spatial transcriptomic techniques enable the mapping of gene expression directly onto histological structures. Spatial transcriptomics can be categorized into (1) RNA capture-based approaches (e.g., 10× Visium, Slide-seq) where tissue sections are placed on slides patterned with spatially barcoded primers that capture RNA molecules and tag them to record their spatial locations; (2) in probe- or imaging-based approaches [e.g., multiplexed error-robust fluorescence in situ hybridization (MERFISH), sequential FISH (seqFISH), Xenium in situ], where fluorescently labeled probes hybridize target transcripts on a tissue cross-section, enabling direct visualization of transcripts locations at high spatial resolution [71]. It allows more precise characterization of tissue diversity, cell identity, and spatially regulated gene expression, offering insights into how transcriptional activity is influenced by cellular neighborhoods and microenvironmental context [2,4]. Recent studies in soybean have demonstrated the power of integrating spatial transcriptomics with scRNA-seq to profile cell-type-specific transcriptional activity within a spatial framework. Spatial RNA-seq across five tissue types, including multiple developmental stages of seeds, not only enabled accurate cell-type annotation but also revealed spatially differentiated sub-cell types [4]. In another study, the combination of Stereo-seq and scRNA-seq identified novel genes associated with nodule development in soybean [5]. Furthermore, integrating scRNA-seq with high-resolution spatial transcriptomic mapping of mature nodules revealed distinct transcriptomic signatures and uncovered the coexistence of multiple *Bradyrhizobium diazoefficiens*-infected cell subpopulations within mature nodules [2]. These advances highlight the potential of spatially resolved multi-omics to deepen our understanding of plant biology and inform targeted crop improvement strategies.

## 3. Developing Single-Cell Legume Atlases to Capture Cellular Diversity and Plant Cell Environmental Responses

### 3.1. Developmental Atlases of Plant Organs: Single-Cell and Spatial Transcriptomes in Legumes

Large-scale single-cell transcriptomic profiling across multiple organs provides a comprehensive understanding of organ-level transcriptional dynamics, reveals novel genetic programs including those conserved among different cell types, and supports the functional characterization of plant genes. Advances in single-cell and spatial transcriptomics have enabled the construction of developmental atlases for non-model crops, including legumes, providing cellular-resolution insights into tissue complexity. For instance, a recent single-nucleus transcriptome atlas of peanut constructed from leaves, stems, hypocotyls, and roots identified distinct cell types, reconstructed developmental trajectories, and highlighted key TFs and interaction networks regulating hypocotyl cell differentiation. Among these TFs, *AhWRKY70* was identified as a potential regulator of stem elongation acting by modulating the auxin and ethylene signaling pathways [65]. In a complementary study by Cui et al. [62] profiled the nuclear transcriptome across different stages of peanut pod development, revealing gravity and light responsive genes involved in pod formation and delineating differentiation trajectories of parenchyma cells. To complement these studies, leaf scRNA-seq isolated from peanut seedlings grown under dark and light conditions uncovered gene networks underlying light-mediated repression of cell division and hormonal regulation of epidermal cell differentiation [63]. Another scRNA-seq study on peanut leaf blades revealed distinct transcriptional states of mesophyll and epidermal cells, suggesting that palisade mesophyll cells may transition into spongy cells and that epidermal cells emerge earlier than primordium cells [57].

**Figure 2:**
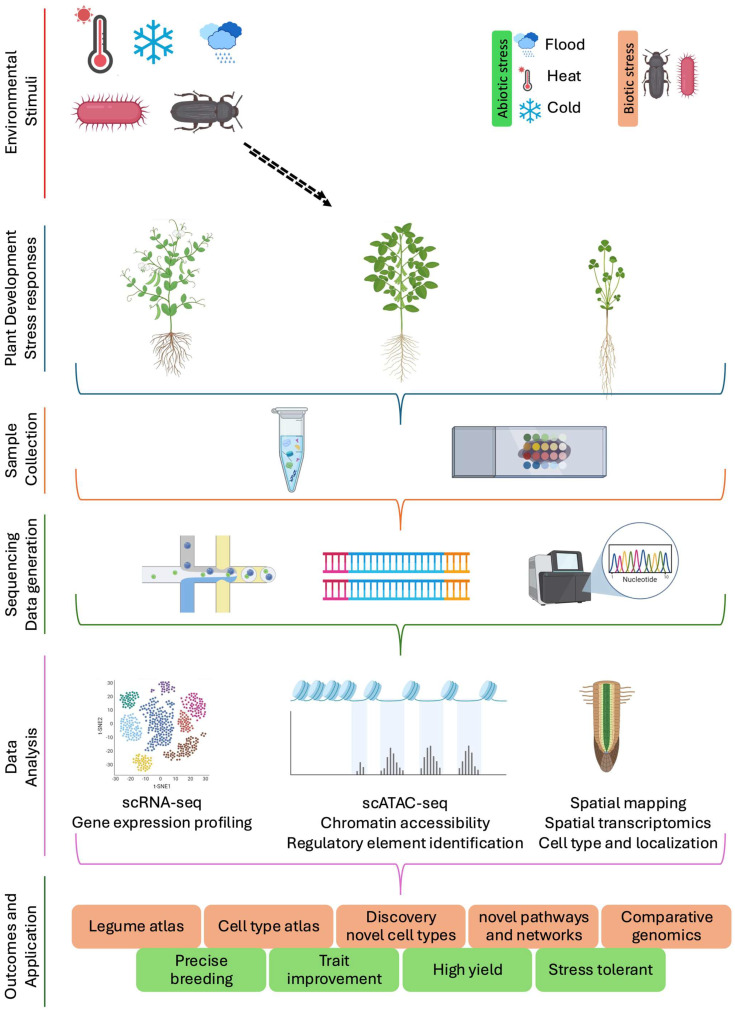
Integrated multi-omics approaches for a comprehensive understanding of plant growth, development, and environmental responses. Samples from different tissues, developmental stages, or stress conditions are processed to generate scRNA-seq, snATAC-seq and spatial transcriptomics data. These datasets are integrated to construct a legume cell atlas that captures gene expression profiles, cell type localization, and regulatory landscapes. Cross-species comparisons of the integrated atlas provide functional and evolutionary insights into legume biology. Collectively, these approaches enable the identification of key regulatory genes and pathways, contributing to the development of resilient crop varieties. The arrow indicates the environmental signal responsed by plants. The orange box highlights applications while green box highlights the future perspectives of single cell transcriptomics. Created with BioRender.com.

In soybean, Zhang et al. [4] recently developed a spatially resolved multi-omic single-cell atlas by integrating scRNA-seq, snATAC-seq, and spatial transcriptomics across various organs, including leaf, hypocotyl, root, nodule, young pod, and seeds. This study identified three transcriptionally distinct sub-cell types in the endosperm and 13 sucrose transporter genes, including *GmSWEET15a* and *GmSWEET10a*, which were co-expressed in late peripheral endosperm and regulated by *GmDOF11* [4]. In another study, researchers developed a whole-soybean single-cell transcriptome atlas, identifying distinct cell clusters across 10 organs by integrating spatial transcriptomics and orthologous gene relationships. TF analysis revealed that co-expressed TF modules are sufficient to define the identities of organs and cells. For example, within the *GmWRKY* family, 20 genes were preferentially expressed in root epidermal clusters (mainly clusters 1, 2, and 4 excluding root hairs) and cortical tissues, while 33 genes showed predominant expression in the root epidermal (cluster 4) and root hair cells (cluster 3) [9]. To complement these resources, Fan et al. [5] developed a scRNA-seq atlas across five soybean organs including the root tip, shoot apex, leaf, stem, and root nodule that led to the characterization of a distinct stem cell niche cluster marked by the expression of *GmPLT4*, *GmPLT1a*, and *GmPLT1b*, which belong to the *GmAP2* TF family and are closely related to *AtPLETHORA* genes, master regulators of root stem cell activity in Arabidopsis [72]. Together, these atlases provide reference frameworks for dissecting transcriptional and epigenetic regulation of organ development in legumes, enabling comparative genomics across species and guiding targeted crop improvement strategies (Figure 2).

### 3.2. Stress and Immunity-Responsive Atlases

Biotic and abiotic environmental factors, such as pathogen attack, drought, heat, and nutrient deficiency, profoundly influence plant growth and productivity. They trigger complex signaling cascades and transcriptional reprogramming. These responses vary between tissues and cell types, reflecting specialized cellular strategies for adaptation [73,74,75,76]. At the initial site of pathogen entry, epidermal and root hair cells rapidly activate pattern-triggered immunity (PTI) through receptors such as *FLS2* and *EFR*, initiating MAP kinase cascades and early defense gene expression [77]. Guard cells also contribute to modulating abscisic acid (ABA) signaling, which induces stomatal closure as a barrier against fungal entry. Meanwhile, epidermal cells upregulate *MYB122* to promote indole glucosinolate production. Inner tissues, including cortical and vascular cells, mount subsequent immune responses involving salicylic acid (SA)-mediated signaling, bursts of reactive oxygen species (ROS), and activation of pathogenesis-related (PR) genes while vascular cells are enriched in nucleotide-binding leucine-rich repeat (NLR) receptors for intracellular immune surveillance [78]. The advent of scRNA-seq technologies has enabled single-cell resolution in dissecting these heterogeneous biotic responses [79,80,81]. For instance, recent studies have used these approaches to uncover cell-type-specific responses to biotic stresses in Arabidopsis leaves, such as infections by *Pseudomonas* and *Colletotrichum* [74,76,78]. Additionally, specialized immune-active cells, such as primary immune responder (PRIMER) cells, have been identified as local hubs for pathogen detection that relay early immune signals to neighboring “bystander” cells, facilitating long-distance defense communication [82]. Other studies in Arabidopsis reported that only subsets of cortical or mesophyll cells strongly activate NLR receptors, defense-related TFs, and genes associated with antimicrobial metabolites, whereas the same type of neighboring cells remain transcriptionally inactive [78,82]. Similar cellular heterogeneity has been observed in Arabidopsis roots, where scRNA-seq analyses have revealed cell-type-specific immune responses, including the induction of triterpenoid biosynthesis pathways in epidermis and procambium cells to balance beneficial and pathogenic microbial interactions [83]. Taken together, these studies on the model species Arabidopsis support the relevance of the use of single cell omics approach to better decipher the programs used by different cell types to respond to environmental stresses.

When considering legumes, in soybean, scRNA-seq has been employed to examine the transcriptional responses of soybean leaf cells to Soybean Mosaic Virus (SMV) infection. The study analyzed the transcriptome of 50,294 leaf protoplasts from infected and control plants and identified 19 distinct cell clusters, providing a high-resolution map of cell-type-specific transcriptional dynamics [10]. The *tau-class glutathione S-transferase* (GSTUs) are key mediators of plant responses to diverse abiotic and biotic stresses, including viral infections. Among the 57 *GSTU* genes, *GmGSTU23* and *GmGSTU24* were strongly upregulated in epidermal, mesophyll, and vascular cells upon SMV infection in soybean. Functional assays in *Nicotiana benthamiana* confirmed their antiviral activity, highlighting these GSTUs as key contributors to soybean defense mechanisms [10]. Of course, mutualistic symbiotic interactions between legumes and soil microbes (i.e., mycorrhization and nodulation) have also been subject to a significant number of scRNA-seq studies (see below).

Although single-cell atlases have revealed extensive cellular diversity in legumes, future research should integrate developmental and stress-responsive datasets to uncover cell states transition. Key questions include: how do different cell types of coordinates signaling and stress responses? What is the level of conservation of these regulatory mechanisms and the role of associated TFs across legumes and other plant species? What is the impact of chromatin accessibility modulating these processes? Moving beyond descriptive cataloguing, legume single-cell research is now positioned to develop predictive models that connect cell-type-specific gene regulation with plant adaptation, ultimately guiding the engineering of stress-resilient and high-yielding crops.

## 4. Symbiosis as a Model: Insights from Nodulation and Mycorrhizal Interactions

### 4.1. Root Nodule Symbiosis: As a Model for Organogenesis and Signaling

Legume nodulation results from the mutualistic symbiotic interaction between plants (mostly from the legume clade) and soil microbes collectively referred to as rhizobia (e.g., *Rhizobium*, *Bradyrhizobium*, *Sinorhizobium*). This process resulted in the formation of nitrogen-fixing nodules. Under low-nitrogen conditions, legumes secrete flavonoids that attract rhizobia. In response, rhizobia release Nod factors (NFs), which are perceived by the root epidermal cells, triggering the curling of root hairs to entrap the bacteria and initiating the formation of an infection thread that enables the infection of the deeper cellular layers of the root. Concurrently, outer cortical cells located near the protoxylem, particularly those corresponding to cortex layer 4, undergo dedifferentiation and cell cycle reactivation to form a nodule primordium, ultimately developing into a mature nodule [84,85]. Nodule organogenesis is an excellent model for studying plant organ development, as it demonstrates how differentiated root cells can re-enter the cell cycle, change identity, and acquire specialized functions. This developmental reprogramming is tightly coordinated by bacterial signals, plant hormones such as cytokinin and auxin gradients, and a complex network of TFs that collectively regulate cortical cell dedifferentiation, primordium initiation, and the spatial organization of mature nodules [12]. Because nodulation involves diverse cell lineages including root hair cells, dividing cortical and pericycle cells, and infected and uninfected nodule cells, each with distinct transcriptional programs. Single-cell transcriptomics has emerged as a powerful tool for dissecting the dedifferentiation and redifferentiation trajectories, and the discovery of novel regulatory genes controlling nodule development.

Focusing on early symbiotic events, Pereira et al. [12] demonstrated that Medicago epidermal root hair and stele sub-cell types participate in symbiotic responses during infection and in regulating nodule proliferation. Consistently, Medicago root hair, cortex, endodermis, and pericycle cells exhibited the strongest differential gene regulation after 48 h of rhizobium inoculation [58], which aligns with global gene expression reprogramming observed in the epidermis and cortex within 30 min of NFs treatment [61], indicating that early infection triggers a multilayered cascade that propagates from the epidermis toward inner cortical tissues. In Lotus, Frank et al. [14] identified and profiled several populations of cells at different stages of the infection process, including infected root hair and cortical cells, nodule cells, and bacteroid-containing cells [14]. Another scRNA-seq study of Lotus roots identified seven major cell types and revealed associated regulatory programs, including those for phytohormones and nodulation-related gene, showing that species-specific nodulation programs still converge on conserved hormone-responsive and infection-responsive pathways across legumes [8].

Later, in soybean nodule, Zhang et al. [4] highlighted the conserved gene regulatory networks underlying soybean-rhizobium symbiosis [4]. Focusing on metabolic and developmental mechanisms, Sun et al. [60] reported that soybean purine biosynthesis genes were differentially expressed across specific nodule cell types, including infected cells, inner cortex, and phloem, highlighting coordinated intercellular metabolic partitioning of the purine biosynthesis pathway. The study also identified crucial roles for cytokinin and its receptor *GmCRE1*, which are essential for proper nodule formation and efficient nitrogen fixation [60]. These results collectively illustrate how metabolic specialization emerges across infected and uninfected domains, reinforcing the view that nodules function as spatially integrated metabolic organs rather than homogeneous structures. To complement these studies, single-cell transcriptomic maps of soybean roots and mature nodules revealed multiple subpopulations of rhizobia-infected cells, including actively nitrogen-fixing and senescing cells. Gene co-expression analyses also revealed the role of specific TFs and regulatory modules to control the infection, nitrogen fixation, and senescence programs in these subpopulations [2]. Similarly, uninfected cells in the soybean nodule specialize into several distinct subgroups which are essential to support the symbiosis [13]. Likewise, single-cell transcriptomic analysis in Medicago revealed that uninfected cells in the nitrogen fixation zone contribute to nitrogen assimilation through asparagine biosynthesis [59]. Similarly, a study in Medicago identified rare cell subtypes with distinct roles in nodule development, showing that uninfected cells differentiate into specialized subtypes mediating nutrient and energy exchange for efficient symbiotic nitrogen fixation, while infected cells comprise subtypes enriched in infection thread and symbiotic nitrogen fixation genes, highlighting their critical roles in rhizobial infection and nodule development [61]. Another single-cell analysis in Medicago also revealed unique transcriptional signatures for each cell type, with extensive intercellular coordination underlying successful nodule initiation [12]. Together, these studies demonstrate that single-cell omics has shifted the nodulation field from describing organ-level events to resolving the fine-scale division of labor among cell types and subtypes, thereby establishing RNS as a premier model for understanding how cellular diversification and intercellular signaling drive organogenesis and plant–microbe interactions.

### 4.2. Root Nodule Development: Lineage Fate and Spatial Patterning

Spatial patterning of nodules is controlled by cell-type-specific gene regulatory networks. Recent single-cell transcriptomics studies have provided single-cell resolution of lineage fate specification during nodulation. In Medicago, trajectory analysis revealed that nodule organogenesis originates from third-layer cortical cells, which transition through cytokinin-responsive states before adopting auxin-enriched transcriptional identities that drive primordium initiation [12]. This transition is followed by activation of key nodulation genes (e.g., *NIN*, *NF-YA1*), coupled with the expression of cytokinin degradation genes, marking a shift from cytokinin-dependent initiation to auxin-mediated primordium growth and meristem specification [12]. Another study revealed that specific subtypes of epidermal and cortical cells in Medicago undergo transcriptional reprogramming in response to NF signaling [61]. Similarly, distinct epidermal and cortical cell subtypes in Lotus roots respond to rhizobia and initiate infection thread formation [14]. In soybean nodules, infected and uninfected cells further diversify into functionally specialized subpopulations that play crucial roles in nutrient exchange and the establishment of effective symbiosis [13]. Future applications of single-cell technologies could be employed to investigate the programs governing the senescence of nodule cells, enabling reconstruction of gene regulatory networks that govern fate transitions during nodule aging and termination of symbiotic nitrogen fixation, and comparing the level of conservation of these programs between legume species; especially when considering the differential developmental patterns of determinate vs. indeterminate nodules.

### 4.3. Early Signaling Genes Shared in Mycorrhizal and Rhizobia Symbiosis

The establishment of both rhizobial nodulation and arbuscular mycorrhizal (AM) symbiosis relies on a conserved signaling cascade, the Common Symbiotic Signaling Pathway (CSSP) [86,87]. This pathway, originally associated with AM symbiosis, was later co-opted by legumes to support rhizobial endosymbiosis [88]. Key components of this pathway include receptor kinases, nuclear calcium channels, and calcium/calmodulin-dependent protein kinases, which ultimately activate transcriptional regulators guiding either nodule organogenesis or arbuscule formation. Comprehensive overviews of CSSP architecture and function are available in several reviews, but these frameworks were largely derived from bulk analyses and therefore could not resolve which specific epidermal or cortical populations deploy conserved versus symbiosis-specific modules [87,89,90]. Recent scRNA-seq studies now enable the dissection of symbiotic gene activity at cellular level. For instance, *MtVPY* required for infection thread progression and arbuscule development Medicago, shows strong co-expression with other endosymbiotic genes in colonized cells. This dual requirement for *VPY* in both infection-thread formation and arbuscule maturation directly supports the idea that early downstream components of CSSP are functionally reused across AMS and RNS rather than operating as two independent modules. Similarly, *MtEXO70i* is specifically expressed in Medicago infected cortical cells, where it regulates the development of infection threads in nodulation and arbuscule branching in arbuscular mycorrhizal symbiosis [88]. Because *EXO70i* operates only in colonized cortical lineages, its expression pattern provides cellular-level evidence that CSSP-mediated signaling bifurcates downstream into symbiosis-specific trafficking pathways despite sharing upstream calcium decoding. According to Liu et al., the strong overlap between genes induced by Myc-LCOs and NFs indicates that NFs can engage AMS signaling components through the common symbiotic pathway [61]. This transcriptional overlap is mechanistically consistent with CSSP-controlled nuclear Ca^2+^ oscillations being a shared activation node, explaining why single-cell profiles from LCO- and NF-treated roots converge on similar cortical subtypes. Therefore, comparative single-cell analyses of roots colonized by rhizobia and arbuscular mycorrhizal fungi within the same host plant have strong potential to uncover cell-type-specific functions and to delineate both conserved and unique regulatory modules underlying these symbioses.

Recent single-cell studies have provided unprecedented resolution of symbiotic processes in legumes; however, many aspects of nodule biology remain unexplored. Key questions include: What is the level of coordination of cell-type-specific programs to support nodule development, bacterial infection, and plant-microbe symbiosis? Can we engineer the genome of rhizobia-infected cells to enhance their nitrogen fixation rate or lifetime? How do TFs and the accessibility of cis-regulatory elements contribute to the regulation of the genetic programs of the nodule cells? It also remains unclear how CSSP is spatially deployed during rhizobial and arbuscular mycorrhizal colonization, as well as how these pathways intersect at the single-cell level. Future research should integrate comparative single-cell omics with chromatin accessibility and spatial datasets, complemented by targeted perturbations such as cell-type-specific CRISPR, to reconstruct developmental trajectories and identify conserved versus species-specific regulatory modules.

## 5. Implementing Functional Genomic Solutions at Cellular Resolution

With single-cell and spatial transcriptomic atlases now available for several legumes, the next challenge is to implement functional genomic strategies at cellular resolution to enhance specific traits. These approaches aim to connect gene expression profiles with chromatin accessibility, regulatory element activity, and TF binding. By integrating high-throughput single-cell omics with perturbation assays and computational modeling, researchers can link descriptive cell atlases to causal inference and predictive modeling of cell identity programs [2,4,14,59]. When integrated with perturbation assays and computational network reconstruction, single-cell data increasingly link molecular signatures to experimentally validated regulatory circuits [12,60].

### 5.1. Deciphering Cell-Type-Specific Gene Expression Landscapes

Single-cell and spatial transcriptomic technologies have enabled the resolution of cell-type-specific expression patterns [55,70,91], frameworks first established in Arabidopsis and maize and now extended to legumes. In soybean, single-cell and spatial transcriptomic atlases resolved distinct root and nodule populations and identified sucrose transporters such as *GmSWEET15a* and *GmSWEET10a* as regulators of seed endosperm development [2,4]. The ability to localize these transporters to specific cell types clarified their functional role in a way that an organ-level profiling could not, given the complex mixture of cell types composing the seed and its endosperm. Work in Lotus also illustrates how transcriptional differences between neighboring cortical cells can foreshadow developmental outcomes. Cellular heterogeneity detected in infection-associated cell clusters [14] later aligned with genetic evidence showing that *LjSYMRKL1* is required for infection thread progression. The correspondence between atlas-resolved cellular states and a precise developmental requirement provides one of the clearest examples of how single-cell data can identify points of vulnerability or regulation within the symbiotic program [14]. Studies in peanut extended this idea in a different direction. Cell-resolved datasets from pods and leaves uncovered gene expression modules shaped by positional and mechanical cues, including responses to light exposure and gravity [31,32]. The spatial separation of these modules across tissues indicates that organ-specific physical environments play a major role in defining cell identity in legumes [62,63]. Importantly, several of these candidate regulators have now been validated experimentally, including soybean sucrose transporters through physiological assays and *LjSYMRKL1* through mutational analysis [4,14]. These studies reveal a common trend: legume single-cell atlases are moving beyond cell-type identification and toward functional dissection of regulatory genes that coordinate nutrient allocation, infection, and organ development. Collectively, these studies illustrate how legume single-cell resources advance from descriptive expression maps to functional genomic frameworks, laying the groundwork for causal inference, predictive modeling, and translational applications in crop improvement.

### 5.2. Chromatin Accessibility and Protein–DNA Interaction Mapping

Cell identity is determined by transcriptional activity and the accessibility of cis-regulatory elements that control TF binding. Studies in Arabidopsis root single cells have demonstrated that cell-type-specific ACRs strongly correlate with expression profiles [70]. This observation was confirmed in a recent study on soybean through the integration of scRNA-seq and snATAC-seq to construct a spatially resolved multi-omics atlas encompassing 316,358 cells across ten organs [4]. Within nodule-infected cells, the study identified 303,199 ACRs, with approximately 40% displaying cell-type specificity. These regions were enriched for DNA binding motifs of key symbiotic regulators, including *NIN* and *NF-YA1* [92], which have functionally conserved roles in nodule organogenesis and the establishment of a successful symbiosis [93,94]. In soybean, the *GmNN1/FT2a–GmNFYA-C* module also regulates nodulation via shoot-to-root signaling [95], and recent multi-omic mapping in this species further supports TF motif enrichment/localization [4]. The convergence between spatial ATAC-defined ACRs and DAP-seq-defined TF binding sites suggests that long-distance signaling modules, such as *FT-NF-YA*, act by modulating chromatin states at infection and cortical reprogramming loci, providing a mechanistic link between systemic cues and local cell-type specification. By linking regulatory element accessibility to key TFs in infection and cortical cells, these studies create a foundation to engineer cell-type-specific regulatory circuits in legume roots and nodules. These efforts include integrating scRNA-seq and snATAC-seq profiles with TF binding datasets from DAP-seq (e.g., 148 soybean TFs [96]) or ChIP-seq, which will allow more precise mapping of TF–DNA interactions. Moving forward, cell-type-resolved chromatin accessibility assays combined with spatial ATAC-seq and DAP-seq datasets will be instrumental in validating predicted TF-DNA interactions in situ. In addition, emerging in situ chromatin profiling can map euchromatin and hetero-chromatin states and reveal chromatin-state heterogeneity at cellular resolution, providing complementary information for refining regulatory annotations [97].

### 5.3. Regulatory Network Inference from Integrated Single-Cell Data

The integration of single-cell transcriptomics, chromatin accessibility profiling, and TF binding data provides a foundation for reconstructing GRNs underlying legume biology. When considering the nodulation process, trajectory inference and RNA velocity analyses have resolved two major developmental trajectories within Medicago nodules: a symbiotic lineage characterized by the stepwise activation of infection and nitrogen fixation genes, and a non-symbiotic branch diverging from distinct meristematic progenitor populations [59]. The bifurcation of these trajectories makes it possible to pinpoint transcriptional decisions that commit cortical cells either toward rhizobial accommodation or toward alternative developmental fates, offering a framework to study how regulatory inputs shape lineage divergence. To explore the regulatory logic underlying such divergent cell states, network modeling approaches, such as Single-Cell regulatory Network Inference and Clustering (SCENIC) and Multi-omics Integration for Network Inference with Accessibility and Chromatin data (MINI-AC) have been applied to predict TF–target interactions in a cell-type-specific manner [98,99]. For example, GRN inference consistently highlights NIN and NF-YA TFs as central hubs in infected cortical cells, confirming their established role in rhizobial entry and infection thread formation [12]. Beyond validating known regulators, these GRNs predict additional TF modules associated with cortical cell proliferation, vascular differentiation, and senescence programs, thereby nominating new candidate regulators for experimental validation [2]. Patterns emerging from these predictions indicate that nodules rely on a layered regulatory architecture, in which infection-related TFs interface with modules controlling cell-cycle progression and vascular function, linking early infection cues to late-stage tissue differentiation. These predictions are strengthened by integrating GRN inference with chromatin accessibility and TF-binding datasets, enabling the construction of cell-type-resolved GRNs in legumes [4,100]. These networks reveal distinct yet interconnected transcriptional circuits across infection, vascular, and fixation zones, illustrating how spatially organized TF activities coordinate nodule development and symbiotic efficiency [14,59]. Such spatial partitioning of regulatory circuits demonstrates that nodules do not rely on a single master regulator but on distributed TF networks whose activity zones correspond to metabolic and developmental demands of each cell type. Collectively, these advances demonstrate that GRN inference provides not only confirmation of canonical symbiotic regulators but also a predictive framework to discover novel transcriptional players shaping legume cell identity and nitrogen-fixing capacity.

### 5.4. Perspectives and Future Directions

Recent advances in single-cell multi-omics have established foundational frameworks for dissecting cellular heterogeneity and regulatory mechanisms in legumes. Progress has been made in three major directions: (i) cell-type-specific expression profiling; (ii) mapping chromatin accessibility and TF–DNA interactions; (iii) inferring GRNs from integrated datasets. Studies on Lotus, Medicago, and soybean have generated high-resolution models that elucidate the developmental and symbiotic regulatory programs driving nodule formation and function [13,14,61]. Future research will benefit from combining spatial transcriptomics, three-dimensional chromatin conformation mapping, DNA methylation, and histone modification data in situ to capture higher-order genome architecture. In parallel, the development of cell-type-specific CRISPR/Cas9 genome editing systems will enable in planta validation of predicted regulatory modules [101]. The conceptual framework of the legume single-cell atlas synthesizes these functional genomic strategies with genetic variation, comparative cell atlases, and translational breeding applications, providing an integrative roadmap for future research and crop improvement.

## 6. Integrating eGWAS and G×E with Cell Atlases: Toward Predictive Breeding

Linking natural genetic variation to cell-type-specific expression traits is essential for translating single-cell discoveries into breeding applications. While single-cell atlases provide unprecedented cellular resolution, they currently lack integration with population-scale genomic datasets. Recent advances in single-cell genomics, combined with expression quantitative trait locus (eQTL) mapping and expression genome-wide association studies (eGWAS), will enable fine-mapping of regulatory variants underlying complex traits at single cell resolution. Integrating single-cell-resolution transcriptomic atlases with eGWAS and genotype–environment (G×E) interaction analyses provide the opportunity to uncover the role of genes controlling a unique plant trait and expressed in specific cell types or that emerge only under particular environmental conditions, thereby refining our understanding of the genetic architecture of complex agronomic traits [102].

### 6.1. Cell-Type–Resolved eQTL and eGWAS Mapping

Combining GWAS with single-cell transcriptomic data enables a precise assessment of the effects of single-nucleotide polymorphisms (SNPs) on gene expression within defined cell types. In humans, the sc-eQTLGen consortium has pioneered efforts to resolve the impact of gene expression variation at the single-cell level on specific traits. For instance, using scRNA-seq of peripheral blood mononuclear cells (PBMCs), this consortium identified hundreds of cell-type-specific cis-eQTLs, including loci active exclusively in certain immune cell subsets, and reconstructed personalized gene regulatory networks across donors [103]. This work demonstrates the feasibility of connecting natural genetic variation to transcriptional regulation within defined cellular contexts. In plants, large-scale eQTL mapping has traditionally relied on bulk RNA-seq data. For example, Xu et al. [104] estimated cell-type proportions in Arabidopsis leaf tissues using computational deconvolution based on single-cell reference profiles, to identify over 15,038 cis-eQTLs, 62.83% of which showed significant cell-type interaction effects. Hence, the authors highlighted the importance of cell-type-aware modeling in capturing context-specific regulatory variation.

In legumes, cell-type-resolved eQTL datasets are missing. However, a recent preprint by Sun et al. [105] combined a soybean root scRNA-seq atlas with GWAS of root system architecture, revealing that gene networks predominantly expressed in the endodermis and metaphloem were associated with root width and lateral root length. Earlier GWAS using the SoySNP50K panel also identified loci controlling root architecture [106]. The convergence between expression domains and trait-associated loci suggests that regulatory variants often act within narrowly defined cellular niches rather than at the whole-organ level, underscoring why bulk-based approaches fail to capture such effects. Together, these studies highlight the emerging potential of coupling high-density genotyping with single-cell datasets to accelerate causal discovery and guide precise breeding for enhanced nodulation, root architecture, and stress adaptation. As high-throughput single-cell genotyping and transcriptomics technologies become more accessible for crop species, we expect that cell-type-specific eQTL and eGWAS frameworks will soon enable fine-scale dissection of regulatory variants, bridging the gap between genotype, cell-type-specific expression patterns, and agronomic phenotypes in legumes. Together, these findings emphasize that integration of single-cell reference maps into population-scale genetic studies will uncover context-specific regulatory variants that cannot be captured by bulk approaches.

### 6.2. Single-Cell Resolution G×E Analysis

G×E interactions often emerge only in specific cell populations under stress, a complexity that bulk analyses cannot resolve. In Arabidopsis, cell-type-specific Ca^2+^ oscillations in the endodermis and pericycle under salt or osmotic stress highlight the heterogeneity of stress signaling [107]. More recently, multi-omics studies identified CBL-interacting protein kinase 23 (CIPK23) and NIN-Like Protein 7 (NLP7) as conserved drought response drivers across cell types [108]. The restricted activation of these pathways within cellular domains indicates that environmental responses are organized at the level of individual cell states rather than entire tissues, making single-cell approaches essential for attributing regulatory variation to its correct cellular context. Although single-cell G×E eQTL studies are lacking in legumes, these examples suggest that regulatory variation underlying nitrogen fixation and stress tolerance likely acts in a context-specific manner. Integrating legume single-cell atlases with GWAS/eGWAS across environments can help uncover stress-induced, cell-type-specific variants, advancing predictive breeding strategies.

### 6.3. Future Directions and Public Platforms

In the future, expanding the current whole-plant soybean single-cell transcriptome atlases to other legume species will be essential [2,4,5]. Complementary resources will also be required, such as multi-environment GWAS repositories, detailed phenotypic panels, and high-resolution genotype datasets. These resources will enable the integration of population genetics with cell-type-specific expression profiles. Equally important are computational platforms capable of handling large-scale, heterogeneous datasets. Cross-species integration tools (e.g., single-cell alignment mapping (SAMap), orthologous marker gene groups (OMGs), and species-agnostic transformers for universal cell embeddings and networks (SATURN)) need to be adapted for legumes to align cell states across diverse genetic backgrounds and environmental conditions. An integrated ecosystem of such datasets and analytical frameworks is especially critical for legumes, where species diversity, genome complexity, and trait-environment interactions often obscure causal relationships when analyses rely solely on bulk-level information. The establishment of open-access portals that host curated single-cell data, GWAS results, and trait-associated networks will allow researchers to perform reproducible and scalable analyses [109,110,111]. By unifying these resources, legume single-cell research can better resolve links between genetic variation and phenotypic outcomes at cellular resolution. This transition will enable the rational design of genome editing and breeding strategies targeting specific cell populations, ultimately improving nodulation efficiency, nutrient uptake, and stress resilience in legume crops.

## 7. Toward an Integrated Legume Single-Cell Biology: Cross-Species Integration and Synthetic Symbiosis

A comprehensive understanding of legume single-cell biology requires not only the accumulation of datasets but also the development of integrative frameworks that reveal conserved regulatory logic across species. The expansion of legume single-cell transcriptomic resources, combined with multi-omics profiling and synthetic biology, provides unique opportunities to dissect cell-type-specific GRNs. Yet, the field remains fragmented, with datasets produced under heterogeneous annotation standards that hinder cross-species comparisons. A unified framework, anchored in standardized cell ontologies, harmonized multi-species atlases, accurate metadata, and transferable synthetic regulatory modules, is essential to foster interoperability and accelerate the design of synthetic symbioses.

### 7.1. Standardizing Cell Ontologies and Annotation Systems

A major limitation of current legume single-cell studies is the absence of standardized cell ontologies. Datasets across species or even within a species often rely on study-specific clustering and marker genes, complicating integration and comparative analyses. Similar challenges in other fields have been addressed through ontology-based frameworks: the OBO Foundry has provided interoperable ontologies for biomedicine, and the Cell Ontology project has established hierarchical, reproducible classifications [112,113]. More recently, the minimum information required to annotate a cell type (MIRACL) standard introduced guidelines for reporting new cell types with essential metadata, enabling alignment with ontology entries [114]. A plant-specific equivalent of MIRACL, tailored to legumes, would enable consistent annotation, reproducibility, and cross-dataset integration. Such a framework would lay the foundation for multi-species legume cell atlases, advancing comparative studies of nodulation and symbiosis.

### 7.2. Cross-Species Atlas Integration and Conserved Regulatory Modules

Cross-species integration of single-cell datasets provides an approach to uncover evolutionarily conserved transcriptional programs and regulatory networks that control legume biology. While large-scale single-cell integration is well established in animal systems, where methods such as SAMap [111] align homologous cell types across distantly related species; similar efforts in plants are only beginning to emerge. Recent single-cell and single-nucleus atlases from legumes, including soybean roots and nodules [2,4] and Lotus [8], have identified conserved cell populations associated with rhizobial infection, nodule primordium initiation, and vascular remodeling. Consistently, Cervantes-Pérez et al. [58] transferred Arabidopsis markers to Medicago to resolve cell identities and early symbiotic responses, underscoring cross-species conservation at the cell-type level [58]. Comparative analyses demonstrate that key TFs, such as NIN and NLP family regulators, play conserved cell-type-specific expression across species. NIN is essential for nodulation even in the non-legume *Parasponia* [115], whereas NLPs mediate nitrate-dependent inhibition of nodulation in both Lotus and Medicago [7,116,117]. Together, these findings suggest the presence of a shared regulatory backbone governing nodulation. In addition to integration-based approaches, a recent framework named OMGs has been proposed to improve cross-species comparison of plant single-cell datasets [110]. By leveraging orthologous marker gene sets instead of relying solely on one-to-one ortholog alignment, OMGs robustly mapped homologous cell identities across 15 plant species, including multiple legumes, and revealed 14 broadly conserved cell-type groups spanning monocots and dicots. This method complements algorithms such as SAMap and provides a scalable solution for large-scale comparative plant single-cell transcriptomics, enabling the identification of conserved regulatory modules underlying developmental and symbiotic processes. Establishing robust pipelines for cross-species single-cell atlas integration by combining methods like SAMap and OMGs with homology-aware alignment algorithms such as SATURN [109] will be crucial for building a unified framework for legume cell identity annotation. These approaches will facilitate the discovery of deeply conserved regulatory circuits, illuminate lineage-specific innovations, and guide the selection or engineering of symbiotic gene modules for translational applications in crop breeding.

### 7.3. Synthetic Symbiosis and Transferable Trait Modules

**Figure 3:**
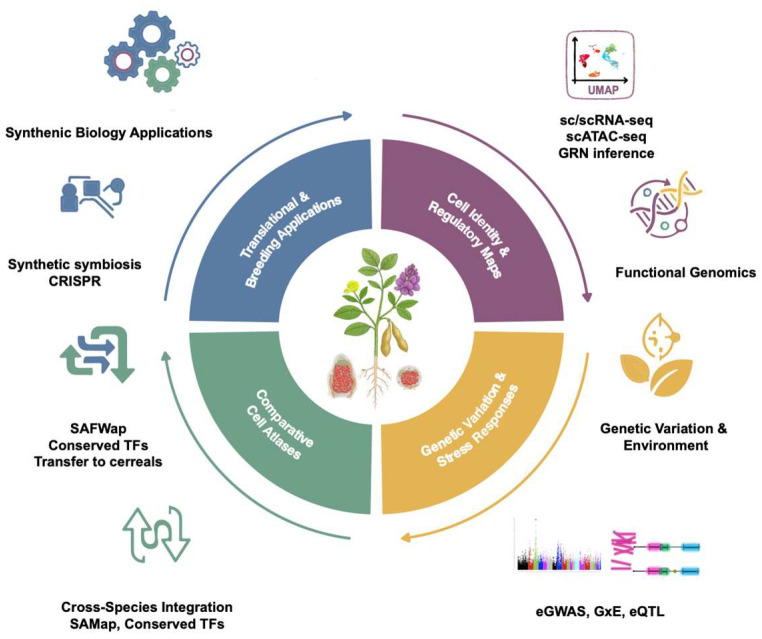
Conceptual framework of the Legume Single-Cell Atlas. The schem illustrates four interconnected research themes: (i) Cell Identity & Regulatory Maps (purple)—defined by scRNA-seq, snRNA-seq, ATAC-seq, and gene regulatory network inference; (ii) Genetic Variation & Stress Responses (yellow)—linking natural variation to cellular programs through eGWAS, G×E, and eQTL analyses; (iii) Comparative Cell Atlases (green)—cross-species integration using SAMap and conserved TFs; (iv) Translational & Breeding Applications (blue)—synthetic symbiosis, CRISPR-mediated engineering, and transfer of regulatory modules to cereals. Together, these directions highlight how single-cell genomics in legumes bridges basic regulatory mechanisms with translational applications in crop improvement.

Synthetic biology offers a compelling opportunity to reconstruct minimal symbiotic circuits and transfer modular regulatory programs from legumes into other plant species. Insights from single-cell atlases have enabled the identification of cell-type-specific regulatory modules that govern rhizobial infection, nodule initiation, and nitrogen-fixing activities [2,12,59,60]. These modules represent programmable elements that could drive symbiotic traits when expressed in heterologous systems. Foundational studies have shown that core nodulation regulators such as NIN and NF-YA TFs are capable, in principle, of triggering nodulation responses in non-legume models, although full functionality remains elusive [115]. In legumes, chromatin accessibility assays combined with transcriptomic analyses have begun to reveal cell-specific regulatory programs underlying symbiosis. For example, time-series (0–24 h) ATAC-seq and RNA-seq analyses in Medicago roots treated with rhizobial lipo-chitooligosaccharides (LCOs) were integrated with dynamic regulatory module networks to predict cis-regulatory elements and TFs, such as EIN3 and ERF1, whose RNAi knockdown reduces nodule numbers, supporting roles in early nodule development [100]. A particularly promising development comes from synthetic microbial-plant communication systems. Specifically, Boo et al. [118] engineered modular interkingdom communication channels in bacteria that sense environmental signals and transmit them to plants via small-molecule messengers, thereby activating plant gene expression. Although this study used Arabidopsis and *Solanum lycopersicum*, the modular signaling concept offers a scalable blueprint for encoding symbiotic triggers into microbial partners for legumes. Looking ahead, combining cell atlas-informed promoter modules with synthetic communication circuits and homology-aligned regulatory networks frameworks such as SAMap, OMGs and SATURN will enable assembly of transferable symbiotic trait modules. These could include multi-gene circuits for nodule initiation, rhizobial infection, and nutrient exchange, optimized to operate in specific cell types. Implemented through genome editing or transgenic expression in legumes or even cereals, such synthetic modules hold potential to reprogram plants for enhanced nitrogen fixation, improve stress resilience, and unlock symbiotic capabilities beyond traditional legume species. Incorporating spatial cell biology in situ helps integrate these single cell approaches by anchoring molecular profiles to their native tissue context. A conceptual framework summarizing these interconnected research directions is illustrated in Figure 3.

Together, these advances mark a shift from descriptive single-cell atlases to the rational engineering of symbiotic traits. Integrating single-cell multi-omics with synthetic biology provides a roadmap toward programmable plant-microbe interactions and next-generation crop design.

## 8. Challenges and Future Directions

Despite remarkable progress in legume single-cell biology, several challenges continue to limit its broader application in plant science and crop improvement. Technical bottlenecks remain prominent: protoplast isolation and nuclei extraction often cause cell damage and low RNA yield, leading to sparse data that compromise the detection of low-abundance transcripts and the recovery of rare or transient states. Recent studies further highlight that even cells of the same type can adopt distinct transcriptional states under stress, for instance, immune-activated versus quiescent mesophyll cells during pathogen infection, or drought-induced mesophyll populations enriched either for ABA- or iron-responsive programs. Such findings illustrate the importance of cell state transitions, yet systematic validation of state-specific regulators and their functional contribution to field-level traits is still lacking.

At the same time, current single-cell atlases remain largely descriptive because of the limited availability of robust cell-type markers, tissue-specific promoters, and high-throughput genome editing tools optimized for legumes. From a computational perspective, the integration of datasets across experiments, genotypes, and environmental conditions remains difficult due to variation in protocols, sequencing depth, and annotation standards. The lack of plant-specific ontologies and standardized metadata further constrains cross-species comparisons and the construction of unified multi-species legume atlases.

Addressing these limitations will require combined efforts. Integrative strategies that merge single-cell and spatial multi-omics with high-resolution mapping approaches (eQTL/eGWAS), advanced computational models of gene regulatory networks, and efficient genome editing platforms are needed to establish predictive pipelines. Such approaches will enable the identification and functional validation of key regulators acting at both the cell-type and cell-state levels. With these advances, single-cell research in legumes can move from descriptive atlases toward predictive and mechanistic models, creating opportunities for rational engineering of nodulation, nutrient uptake, and stress resilience, and ultimately contributing to sustainable crop improvement.

## Data Availability

All data are included in this article.

## Abbreviations

The following abbreviations are used in this manuscript:

ABA	Abscisic acid
ACRs	Accessible chromatin regions
CSSP	Common Symbiotic Signaling Pathway
eGWAS	Expression genome-wide association studies
eQTL	Expression quantitative trait locus
FACS	Fluorescence-activated cell sorting
GRNs	Gene regulatory networks
GSTUs	Tau-class glutathione S-transferase
LCM	Laser capture microdissection
LCO	Lipo-chitooligosaccharides
MIRACL	Minimum information required to annotate a cell type
NF	Nod factor
NLR	Nucleotide-binding leucine-rich repeat
OMGs	Orthologous marker gene groups
PR	Pathogenesis-related
PRIMER	Pathogen-Responsive Immune Relay
PTI	Pattern-triggered immunity
QC	Quiescent center
RNS	Root nodule symbiosis
RNA-seq	RNA sequencing
ROS	Reactive oxygen species
SA	Salicylic acid
SAMap	Single-cell alignment mapping
SATURN	Species-agnostic transformers for universal cell embeddings and networks
sc/snRNA-seq	Single-cell/single-nucleus RNA sequencing
SMV	Soybean Mosaic Virus
snATAC-seq	Single-nucleus Assay for Transposase-Accessible Chromatin using sequencing
TFs	transcription factors
